# Dimensionen von Personenzentrierung in der Versorgung bei Schwangerschaftsabbruch – ausgewählte Ergebnisse der CarePreg Studie

**DOI:** 10.1007/s00103-024-03990-7

**Published:** 2024-12-10

**Authors:** Jördis Zill, Anja Lindig

**Affiliations:** Institut und Poliklinik für Medizinische Psychologie, Universitätsklinikum Hamburg-Eppendorf (UKE), Martinistraße 52, 20246 Hamburg, Deutschland

**Keywords:** Patientenorientierung, Schwangerschaftsabbruch, Ungewollte Schwangerschaft, Qualitative Forschung, Versorgungsforschung, Patient-centered care, Abortion, Unwanted pregnancy, Qualitative research, Healthcare services research

## Abstract

Personenzentrierung ist ein Grundprinzip der Gesundheitsversorgung in Deutschland. Das Konzept stellt die Präferenzen, Bedarfe und Werte von Versorgten in den Mittelpunkt. Studien zeigen, dass unbeabsichtigt Schwangere, die den Wunsch haben, die Schwangerschaft abzubrechen, in der Versorgung mit gesetzlichen Regelungen, Stigmatisierungen sowie ethischen und moralischen Bedenken von Behandelnden konfrontiert sein können. In Deutschland ist die Umsetzung von Personenzentrierung in der Versorgung von unbeabsichtigt Schwangeren kaum erforscht. Ziele der CarePreg-Studie (Laufzeit 11/2020 bis 07/2024) waren es, die Personenzentrierung in der psychosozialen und medizinischen Versorgung (1) aus Perspektive von Expert:innen aus der Versorgung als auch (2) von Personen mit einer unbeabsichtigten Schwangerschaft und einem Schwangerschaftsabbruch zu evaluieren und (3) Handlungsempfehlungen abzuleiten. Zur Durchführung der Studie wurde ein Ansatz aus qualitativen und quantitativen Forschungsmethoden gewählt.

In diesem Beitrag wird die Methodik der CarePreg-Studie vorgestellt und über die Ergebnisse von 2 Workshops mit 18 Expert:innen aus der 1. Phase der Studie berichtet. Die Workshopteilnehmenden stammten aus der psychosozialen und medizinischen Versorgung von unbeabsichtigt Schwangeren. Personenzentrierung wurde von ihnen als höchst relevant für die Versorgung betrachtet. Hervorgehoben wurden folgende Dimensionen von Personenzentrierung: „Zugang zur Versorgung“, „persönlich angepasste Informationen“ und „gleichberechtigte Zusammenarbeit und Beteiligung an Entscheidungen“. Barrieren durch die Stigmatisierung von Schwangerschaftsabbrüchen und von Versorgenden in diesem Bereich sowie durch die aktuellen rechtlichen Rahmenbedingungen wurden diskutiert.

## Abstract (en)

Person-centered care is a fundamental principle of healthcare in Germany. The concept focuses on the preferences, needs, and values of individuals in the healthcare system. Studies show that unintentionally pregnant women seeking to terminate their pregnancy may encounter legal regulations, stigmatization, and ethical and moral concerns from healthcare providers. In Germany, the implementation of person-centered care in the care of unintentionally pregnant women has been scarcely researched. The goals of the CarePreg study (running from November 2020 to July 2024) were to evaluate person-centered care in psychosocial and medical care (1) from the perspective of healthcare providers as well as (2) from the perspective of women experiencing an unintended pregnancy and abortion, and (3) to derive recommendations for the current care situation. A mixed-methods approach was chosen for the study.

This article presents the methodology of the CarePreg study and reports on the findings from two workshops involving 18 experts as part of the first study phase. Participants in the workshops were professionals from psychosocial and medical services for individuals with unintended pregnancies. They considered person-centered care highly relevant to abortion services, emphasizing dimensions such as “access to care,” “personalized information,” and “equal collaboration and involvement in decision-making.” Barriers to person-centered care discussed included the stigmatization of abortion and those providing related services as well as the current legal framework.

## Hintergrund

Im Jahr 2022 veröffentlichte die Weltgesundheitsorganisation (WHO) eine überarbeitete Leitlinie zur Versorgung von Schwangerschaftsabbrüchen, die unabhängig von den rechtlichen und politischen Unterschieden zwischen Ländern als Empfehlung gilt [1]. Im selben Jahr wurde in Deutschland die S2k-Leitlinie „Schwangerschaftsabbruch im ersten Trimenon“ der Arbeitsgemeinschaft der Wissenschaftlichen Medizinischen Fachgesellschaften (AWMF) veröffentlicht [2]. Die Leitlinien betonen die Notwendigkeit eines respektvollen und würdevollen Umgangs mit der Schwangeren sowie die Bereitstellung aller relevanten Informationen für eine selbstbestimmte Entscheidung. In einem partizipativen Prozess sollten die Beratenden und Behandelnden die persönlichen Werte, kulturellen Hintergründe und Lebensumstände der Schwangeren berücksichtigen und auf ihre emotionalen und psychischen Belastungen eingehen. Die Qualität der Versorgung sollte effektiv, effizient, zugänglich, personenzentriert, gerecht und sicher sein.

Die Inhalte der Leitlinien stehen somit im Einklang mit dem Konzept der *Personenzentrierung,* welches auch als zentrales Qualitätskriterium im deutschen Gesundheitswesen gilt [3, 4]. Im Sinne der Personenzentrierung leiten die Präferenzen, Bedürfnisse und Werte des Individuums die Entscheidungen in der Versorgung und die Beziehung zwischen Versorgenden und Versorgten [5].

Auf Basis einer systematischen Übersichtsarbeit von Definitionen zu Personenzentrierung haben Scholl et al. [14] das „Integrative Modell der Personenzentrierung“ entwickelt, das zunächst aus 15 Dimensionen bestand, die in 3 Gruppen unterteilt werden können: *Grundprinzipien, förderliche Faktoren* und *Aktivitäten* [5]. In einer folgenden Studie zur Perspektive von Patient:innen auf das Integrative Modell der Personenzentrierung wurde „Patientensicherheit“ als 16. Dimension ergänzt ([6]; Tab. 1).

**Tab. 1:** Die 16 Dimensionen der Personenzentrierung nach dem „Integrativen Modell der Personenzentrierung“ (leicht modifiziert nach [5] und [6])

Nr.	Dimension der Personenzentrierung
*Grundprinzipien*	
1	Personenzentrierte Merkmale der Beratenden/Behandelnden
2	Vertrauensvolles Miteinander
3	Einbezug der Lebensumstände
4	Einzigartigkeit jeder Person
*Förderliche Faktoren*	
5	Zugang zur Versorgung
6	Gute Planung der Beratung
7	Zusammenarbeit der Beratenden/Behandelnden
8	Einbezug ergänzender Angebote
9	Angemessene Kommunikation
*Aktivitäten*	
10	Persönlich angepasste Informationen
11	Gleichberechtigte Zusammenarbeit und Beteiligung an Entscheidungen
12	Einbezug von Familie und Freund:innen
13	Aktivierung von Personen in der Beratung/Behandlung
14	Unterstützung des psychischen Wohlbefindens
15	Unterstützung des körperlichen Wohlbefindens
16	Patient:innensicherheit

Laut WHO-Leitlinie gehören zu den entscheidenden Aspekten der Personenzentrierung in der Versorgung bei Schwangerschaftsabbrüchen eine informierte Entscheidung, Vertraulichkeit und der Schutz der Privatsphäre in der Gesundheitsfürsorge sowie der Zugang zu legalen und erschwinglichen Versorgungsleistungen [1]. Diese lassen sich im Integrativen Modell der Personenzentrierung den Dimensionen „gleichberechtigte Zusammenarbeit und Beteiligung an Entscheidungen“, „vertrauensvolles Miteinander“, „Patient:innensicherheit“ und „Zugang zur Versorgung“ einordnen [5].

Im breiteren Kontext der reproduktiven Gesundheitsversorgung zeigt sich, dass Personenzentrierung die medizinischen Ergebnisse für Frauen verbessern kann [7]. Aufgrund restriktiver gesundheitspolitischer Regelungen und sozialer Stigmatisierung wurde das Konzept der Personenzentrierung in der Versorgung bei Schwangerschaftsabbruch bzw. in der Gesundheitsversorgung von Frauen mit unbeabsichtigter Schwangerschaft bisher kaum umgesetzt [8].

Mit wenigen Ausnahmen [9, 10] wurden in internationalen Studien hauptsächlich die Erfahrungen von Frauen mit Schwangerschaftsabbrüchen mit einem Schwerpunkt auf der allgemeinen Zufriedenheit mit der Versorgung und nicht auf Dimensionen der Personenzentrierung untersucht [8, 11].

In einer Übersichtsarbeit von Doran et al. [12], die 38 Artikel umfasste, beschrieben Frauen, die einen Schwangerschaftsabbruch vornehmen lassen wollten, folgende Barrieren für die Dimension des „Zugangs zur Versorgung“ zum Schwangerschaftsabbruch: Entfernung zu Versorgungseinrichtungen und Verfügbarkeit von Versorgungseinrichtungen, negative Einstellungen des Personals und hohe Kosten [12]. Diese Übersichtsarbeit ist auch eine der wenigen internationalen Studien, die die Einstellungen und Erfahrungen von Versorgenden in Hinblick auf Leistungen zum Schwangerschaftsabbruch berücksichtigt. Aus ihrer Perspektive werden folgende Barrieren in der Versorgung beschrieben: ethisch und moralisch ablehnende Haltung von medizinischem Fachpersonal gegenüber Schwangerschaftsabbrüchen; mangelnde Ausbildung des Fachpersonals; zu wenige Ärzt:innen, die Schwangerschaftsabbrüche anbieten; Mobbing durch das Personal und unzureichende Ressourcen [12].

In einer neueren Studie über die Erfahrungen von Frauen mit der Versorgung bei einem Schwangerschaftsabbruch in den Niederlanden wurden mehrere Barrieren für den Zugang zur Versorgung genannt, darunter die Tabuisierung und Stigmatisierung von Schwangerschaftsabbrüchen im Gesundheitswesen, eingeschränkter Zugang für gesellschaftlich marginalisierte Gruppen und Schwierigkeiten für Frauen, in der Gesellschaft offen über einen Schwangerschaftsabbruch zu sprechen [13]. Andere Studien verweisen auf die Belastung der Frauen, die für einen Schwangerschaftsabbruch längere Strecken zurücklegen oder das Land verlassen müssen [14, 15].

In Deutschland ist die Datenlage zu den Erfahrungen von Frauen mit einem Schwangerschaftsabbruch und von Fachkräften des Gesundheitswesens mit der Betreuung von Schwangerschaftsabbrüchen sehr begrenzt [16–18] und die Qualität der Versorgung von Schwangerschaftsabbrüchen wurde bisher nicht im Hinblick auf die Umsetzung der Personenzentrierung analysiert. Das Bundesministerium für Gesundheit (BMG) hat dazu im Jahr 2019 Forschungsgelder über 5 Mio. € im Rahmen eines Förderschwerpunkts zur Untersuchung „der psychosozialen Situation und dem Unterstützungsbedarf von Frauen mit ungewollter Schwangerschaft“ bereitgestellt. In diesem Rahmen erhielten 3 Forschungsprojekte eine Förderung: „Erfahrungen und Lebenslagen ungewollt Schwangerer – Angebote der Beratung und Versorgung (ELSA)“, „Die medizinische Versorgungssituation zur Durchführung eines Schwangerschaftsabbruchs im Krankenhaussektor in Deutschland (MedVersKH)“ sowie das Projekt CarePreg („Betroffenenzentrierung von Versorgungs- und Unterstützungsangeboten für Frauen mit ungewollter Schwangerschaft“).

Das Projekt CarePreg (Laufzeit 11/2020 bis 07/2024) hatte 3 Ziele:

1. qualitative Untersuchung der Relevanz und Umsetzung von Dimensionen von Personenzentrierung in der psychosozialen und medizinischen Versorgung sowohl aus der Perspektive von Expert:innen aus der Versorgung als auch von ehemals Betroffenen einer unbeabsichtigten Schwangerschaft,
2. quantitative Evaluation von Personenzentrierung aus der Perspektive von aktuell Betroffenen einer unbeabsichtigten Schwangerschaft mit dem Wunsch nach einem Schwangerschaftsabbruch und
3. Ableitung von Handlungsempfehlungen für die Versorgung.

In dem vorliegenden Bericht wird zunächst ein Überblick zur Methodik der CarePreg-Studie gegeben und daraufhin ausgewählte Ergebnisse zur Perspektive von Expert:innen auf die Relevanz und aktuelle Umsetzung von Dimensionen von Personenzentrierung in der Versorgung bei Schwangerschaftsabbruch vorgestellt.

Eine detailliertere Darstellung der Methodik der CarePreg-Studie findet sich im bereits veröffentlichten Studienprotokoll [19], eine detaillierte Ergebnisdarstellung zu den Expert:innenworkshops findet sich in der Publikation von Lindig et al. [20].

## Die CarePreg-Studie

**Abb. 1:**
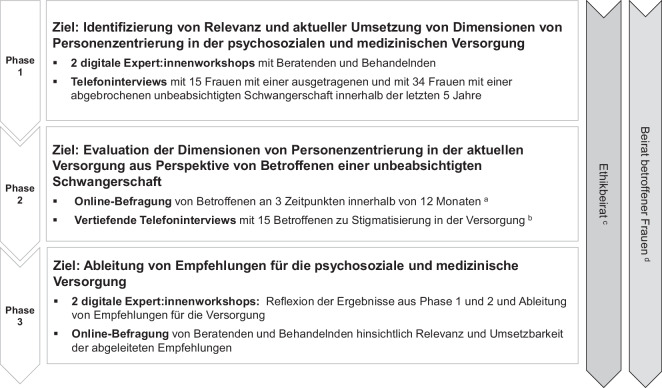
Studiendesign der CarePreg-Studie (eigene durch die Autorinnen für eine andere Publikation erstellte Abbildung [19], Lizenz „Creative Commons Attribution 4.0 International“ [32] für die vorliegende Publikation durch die Autorinnen übersetzt). ^a^Einschluss von Frauen, die sich für den Abbruch oder die Austragung der Schwangerschaft entschieden haben, ^b^Teilnehmende haben sich für den Abbruch der Schwangerschaft entschieden, ^c^2 Vertreterinnen des Beirats betroffener Frauen, 3 Expertinnen aus der Beratung/gynäkologischen Praxis, 2 ethische Beraterinnen, ^d^6 Frauen, die innerhalb der letzten 5 Jahre unbeabsichtigt schwanger waren (Schwangerschaft ausgetragen oder abgebrochen)

Die Studie mit einer Laufzeit von insgesamt 45 Monaten gliederte sich in 3 Studienphasen, in denen jeweils eines der bereits beschriebenen Studienziele verfolgt wurde (Abb. 1). Es wurde ein sequenzielles, exploratives Studiendesign mit einer Kombination aus qualitativen und quantitativen Methoden gewählt [19].

Als theoretische Grundlage der CarePreg-Studie wurde das in der Einleitung beschriebene Integrative Modell der Personenzentrierung mit seinen 16 Dimensionen verwendet [5, 6, 21]. Die Studie wurde begleitet von einem Beirat, bestehend aus 6 Frauen, die selbst eine unbeabsichtigte Schwangerschaft erlebt haben (eine Frau hatte die Schwangerschaft ausgetragen und 5 Frauen hatten die Schwangerschaft abgebrochen). Um die Betroffenen zu beteiligen, fanden regelmäßig Treffen statt, bei denen sowohl die Gestaltung von Studienmaterialien (z. B. Fragebögen), die Gewinnung von Teilnehmenden als auch Fragestellungen und Ergebnisse diskutiert wurden [22].

Entsprechend den Vorgaben des Förderschwerpunktes des BMG wurden in alle Studienphasen nur Personen einbezogen, welche eine Schwangerschaftsabbruchs-Beratungsregelung (nach § 218a Absatz 1 StGB [23]) wünschten oder durchführen ließen. Des Weiteren wurde die Studie durch einen ethischen Beirat begleitet, bestehend aus 2 Personen aus der klinischen Ethik, 1 Beraterin, 1 Gynäkologin sowie 2 Frauen aus dem Beirat der betroffenen Frauen.

## Expert:innenworkshops zur Diskussion der Relevanz und aktuellen Umsetzung von Personenzentrierung in der Versorgung

### Methodik der Expert:innenworkshops

Für die erste Phase der CarePreg-Studie wurde ein qualitativer Forschungsansatz gewählt, um das Forschungsfeld der Personenzentrierung in der Versorgung bei Schwangerschaftsabbruch zunächst in seiner Vielschichtigkeit zu explorieren. Dazu wurden Expert:innen aus der Versorgung zu 2 digitalen Workshops eingeladen. Ziel dieser Methodik war es, einen Einblick in die Versorgungspraxis zu erhalten und das Forschungsfeld klarer zu definieren.

Bei den insgesamt 18 Expert:innen handelte es sich um 8 Ärzt:innen, die Schwangerschaftsabbrüche durchführen, 9 Berater:innen aus der Schwangerschaftskonflikt- oder Schwangerenberatung sowie eine Juristin aus dem Vorstand einer deutschlandweiten staatlich anerkannten Beratungsstelle. Soziodemografische Daten der Teilnehmenden sind Tab. 2 zu entnehmen. Die Teilnehmenden wurden sowohl über die kooperierenden Einrichtungen der CarePreg-Studie gewonnen als auch über die Liste der Bundesärztekammer zu Ärzt:innen, die Schwangerschaftsabbrüche durchführen, eingeladen.

**Tab. 2:** Soziodemografische Daten von Teilnehmenden der Expert:innenworkshops (eigene durch die Autorinnen für eine andere Publikation erstellte Tabelle [20], Lizenz „Creative Commons Attribution 4.0 International“ [32] für die vorliegende Publikation durch die Autorinnen übersetzt.)

		*n*	*%*
**Anzahl der Teilnehmenden**		**18**	**100**
*Alter* ^a^	18–29 Jahre	1	5,5
	30–39 Jahre	3	16,7
	40–49 Jahre	3	16,7
	50–59 Jahre	5	27,8
	60–69 Jahre	4	22,2
	70–79 Jahre	1	5,5
	Fehlende Werte	1	5,5
*Geschlecht*	Weiblich	16	88,9
	Männlich	1	5,5
	Fehlende Werte	1	5,5
*Muttersprache*	Deutsch	17	94,4
	Fehlende Werte	1	5,5
*Konfession*	Römisch-katholisch	2	11,1
	Evangelisch	5	27,8
	Konfessionslos	10	55,5
	Fehlende Werte	1	5,5
*Aktuelle Profession*^b^	Gynäkolog:in	8	44,4
	Berater:in (Sozialarbeiter:in)	7	38,9
	Berater:in (Psycholog:in)	2	11,1
	Rechtsanwält:in im Vorstand einer Beratungsstelle	1	5,5
*Größe des Arbeitsortes*	< 5000 Einwohner:innen	1	5,5
	< 100.000 Einwohner:innen	2	11,1
	≥ 100.000 Einwohner:innen	12	66,7
	Fehlende Werte	3	16,7
*Wissen über Personenzentrierung*	Gar nicht	2	11,1
	Kaum	1	5,5
	Etwas	4	22,2
	Gut	7	38,9
	Sehr gut	2	11,1
	Fehlende Werte	2	11,1
*Wissen über unbeabsichtigte Schwangerschaft*	Gar nicht	0	0,0
	Kaum	0	0,0
	Etwas	0	0,0
	Gut	5	27,8
	Sehr gut	11	61,1
	Fehlende Werte	2	11,1
*Arbeitserfahrung*	Mittelwert (SD)	22,62 (12,81)	
	Bereich in Jahren	2–40	
	Fehlende Werte	1	

Ein:e Teilnehmer:in hat keine soziodemografischen Daten angegeben und wurde als fehlender Wert in Tab. 1 vermerkt (Ausnahme ist die Rubrik „aktuelle Profession“), welche für alle Teilnehmenden bekannt war; ^a^das Alter wurde in Kategorien von jeweils 10 Jahren innerhalb des erwerbsfähigen Altersbereichs erhoben, um eine Beschreibung der Stichprobe ohne Gefährdung der Anonymität zu ermöglichen; ^b^mehr als eine Antwort möglich

*SD* Standardabweichung

Jeder Workshop bestand aus 2 Teilen und dauerte insgesamt 3,5 h. Im ersten Teil des Workshops erhielten die Teilnehmenden eine Einführung in den Hintergrund der CarePreg-Studie sowie in das Integrative Modell von Personenzentrierung, einschließlich der 16 Dimensionen [5]. Für den 2. Teil der Workshops wurden die Teilnehmenden in 2 Gruppen aufgeteilt und die Dimensionen aus dem beschriebenen Modell der Personenzentrierung nacheinander vorgestellt und folgende Fragen zur Diskussion gegeben: „Welche Bedeutung hat diese Dimension für Ihre Tätigkeit?“ „Welche Aspekte sind Ihrer Meinung nach besonders relevant für eine unbeabsichtigt schwangere Frau?“ „Wie sehen Sie die Umsetzung dieser Dimension in der Versorgung in Deutschland umgesetzt?“ „Welche Aspekte sollten ergänzt werden?“

Der 2. Teil der Workshops wurde jeweils per Audioaufnahmegerät aufgenommen und später transkribiert. Die Transkripte wurden mittels qualitativer Inhaltsanalyse ausgewertet [24, 25]. Dazu wurden zunächst deduktive Hauptkategorien entsprechend den 16 Dimensionen des Integrativen Modells der Personenzentrierung über alle Inhalte der Workshops definiert und während des Kodierungsprozesses wurden induktive Haupt- und Unterkategorien ergänzt.

### Ergebnisse der Expert:innenworkshops

Alle 16 Dimensionen des Integrativen Modells der Personenzentrierung wurden in den Expert:innenworkshops als relevant für die Versorgung bei unbeabsichtigter Schwangerschaft beschrieben. Entsprechend wurden 16 Hauptkategorien gebildet und zusätzlich 31 Unterkategorien. Außerdem wurden 4 neue Hauptkategorien gefunden:

1. „Corona-Pandemie“,
2. „politische und rechtliche Aspekte“,
3. „Stigmatisierung von Betroffenen“ und
4. „Stigmatisierung von Beratenden/Behandelnden“.

Diese Hauptkategorien konnten nicht den 16 Dimensionen des Modells zugeordnet werden und wurden daher auf einer übergeordneten Makroebene angeordnet.

**Abb. 2:**
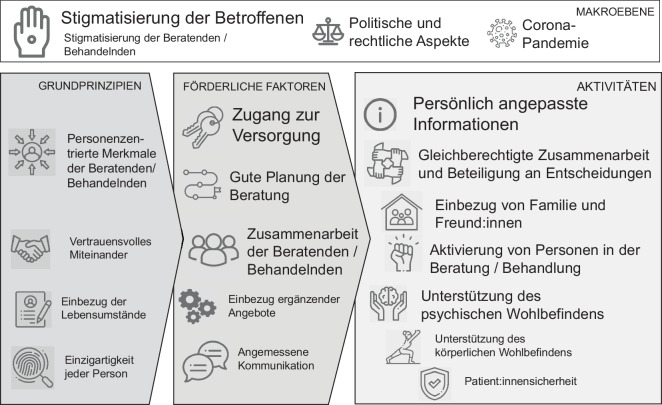
Deduktive Hauptkategorien beschrieben auf Basis des „Integrativen Modells der Personenzentrierung“ [5, 6] sowie induktiv entwickelte Hauptkategorien auf der Makroebene (eigene durch die Autorinnen für eine andere Publikation erstellte Abbildung [20], Lizenz „Creative Commons Attribution 4.0 International“ [32] für die vorliegende Publikation durch die Autorinnen übersetzt). Anmerkungen: Eine größere Schrift bedeutet, dass diese Kategorie von den Teilnehmenden besonders viel und intensiv diskutiert wurde bzw. als besonders relevant für die Versorgung beschrieben wurde

Für einen Überblick über alle 16 Dimensionen und die 4 neuen Hauptkategorien siehe Abb. 2. Eine vollständige Übersicht über das Kategoriensystem und entsprechende Beispielzitate sind in der Ergebnispublikation [20] zu finden.

Im Folgenden werden die Hauptkategorien (Dimensionen von Personenzentrierung) und eine Auswahl der Unterkategorien (kursiv dargestellt) ausführlicher in ihren Inhalten beschrieben. Die Ergebnisse der qualitativen Inhaltsanalyse werden zusammengefasst und entsprechend der Aufteilung des Modells in die 3 Bereiche „Grundprinzipien“, „förderliche Faktoren“ und „Aktivitäten“ von Personenzentrierung sowie die Kategorien der Makroebene dargestellt.

#### Grundprinzipien

*Personenzentrierte Merkmale der Beratenden/Behandelnden und vertrauensvolles Miteinander.* Ein respektvolles und wertschätzendes Verhalten von Versorgenden im Umgang mit Frauen mit unbeabsichtigter Schwangerschaft wurde als entscheidende personenzentrierte Eigenschaft von Versorgenden und als Basis für ein gutes Vertrauensverhältnis genannt. Dies bedeutet innerhalb einer wertfreien Beratung oder Behandlung, Raum für die individuellen Bedürfnisse der Frau zu schaffen und sie darin ernst zu nehmen. Die dieser Dimension zugehörige Unterkategorie *Kompetenz und Informiertheit* wird laut den Teilnehmenden durch nicht immer ausreichendes Wissen zu den Methoden eines Schwangerschaftsabbruchs aufseiten von Gynäkolog:innen, welche selbst keine Abbrüche anbieten, gekennzeichnet. Gründe für mangelnde Kompetenz und Informiertheit lägen auch in der *mangelnden Ausbildung von Gynäkolog:innen im Studium,* weshalb die Teilnehmenden sich für eine verpflichtende Integration der Praxis von Schwangerschaftsabbrüchen in das medizinische Curriculum aussprachen.

##### Einzigartigkeit jeder Person.

Zudem müsse jeder Frau als einzigartige Person mit individuellen Bedürfnissen und Wünschen, insbesondere in Bezug auf ihre Schwangerschaft, ihren Bedarf an mehr oder weniger Informationen und ihre Präferenz für eine Methode zum Abbruch, begegnet werden.

##### Einbezug der Lebensumstände.

Dazu gehöre auch die Berücksichtigung der Lebensumstände der Frau, insbesondere in der Beratung. Nach Möglichkeit sollte nach der *finanziellen und beruflichen Situation* der Frau, einer möglichen *Partnerschaft, nach dem sozialen Umfeld* sowie nach dem *kulturellen und religiösen Hintergrund* gefragt werden. Dies wurde als wichtig beschrieben, weil z. B. die finanzielle Situation die Wahl der Methode zum Abbruch oder ein Mangel an Unterstützung durch das soziale Umfeld die Entscheidung über die Schwangerschaft beeinflussen könnte.

#### Förderliche Faktoren

##### Angemessene Kommunikation.

Die Teilnehmenden beschrieben als Merkmale einer angemessenen Kommunikation von Versorgenden die Schaffung einer vertrauensvollen Atmosphäre, die sachliche und neutrale Vermittlung von Informationen sowie das Adressieren von individuellen Ängsten, Sorgen oder anderen Anliegen der Frauen.

##### Einbezug ergänzender Angebote.

Wichtig sei auch der Einbezug von zusätzlichen Angeboten in die Versorgung, z. B. in der Nachsorge. Hier gäbe es derzeit jedoch einen Mangel an spezifischen psychologischen Beratungsangeboten für Frauen mit unbeabsichtigter Schwangerschaft oder nach einem Schwangerschaftsabbruch.

##### Zugang zur Versorgung.

Die am intensivsten diskutierte Dimension war der Zugang zur psychosozialen und medizinischen Versorgung. Hier wurde der zeitnahe und niedrigschwellige Zugang zu Schwangerschaftsabbrüchen als zentral für eine personenzentrierte Versorgung genannt. Es wurden große regionale Unterschiede in der Qualität der Versorgung beschrieben, besonders eingeschränkt sei diese in ländlichen Gebieten und katholisch geprägten Regionen im Süden Deutschlands. Nach der derzeitigen Regelung stellen sowohl die Kosten zur Selbstübername als auch der Aufwand für eine mögliche Kostenübernahme Barrieren zur Versorgung dar. Als wichtige Bestandteile des Zugangs zur Versorgung wurden genannt: der Zugang zu *Informationen über die Methoden zum Abbruch,* die Möglichkeit der *Wahl der Methode für den Abbruch* und die *Information zu Praxen, die Abbrüche durchführen*. Ein *telefon- oder videobasiertes Beratungsangebots* wurde als gute Möglichkeit genannt, Anfahrtswege und andere organisatorische Hindernisse zur Wahrnehmung des Beratungstermins zu verringern. Zudem empfahlen einige Expert:innen das Angebot der *telemedizinische Durchführung des Schwangerschaftsabbruchs* zu Hause zu erweitern, um den Zugang zu erleichtern. Hier gäbe es derzeit noch zu wenig Wissen von Versorgenden, einen Mangel an Vertrauen in die Gewissenhaftigkeit der Frauen bei der Anwendung dieser Methode und eine unklare Rechtslage.

##### Zusammenarbeit der Beratenden/Behandelnden.

Weiter wurde über die Kommunikation und Zusammenarbeit zwischen unterschiedlichen Versorgungsstellen diskutiert und es wurden Schwierigkeiten genannt, wenn zum Beispiel Krankenhäuser die Versorgung bei Schwangerschaftsabbruch oder sogar die Nachsorge aus *ethischen oder moralischen Bedenken* ablehnen.

##### Gute Planung der Beratung.

Eine gute Zusammenarbeit und Informationsaustausch sind auch ausgesprochen wichtig für die Kontinuität und gute Planung der Behandlung. Dazu gehört auch ausreichend Zeit für Beratung und medizinische Konsultationen. Unabhängig von ihrer Entscheidung sollten den Frauen Nachsorgetermine für Beratung und medizinische Versorgung angeboten werden, bei denen auch andere relevante Themen wie Verhütung besprochen werden.

#### Aktivitäten

##### Persönlich angepasste Informationen.

Persönlich angepasste Informationen zum Schwangerschaftsabbruch wurden von den Teilnehmenden als ausgesprochen wichtig für eine personenzentrierte Versorgung befunden. Die Teilnehmenden diskutierten, dass *Manipulation oder (bewusste oder unbewusste) Fehlinformation* von Frauen durch Versorgende immer wieder in der Praxis vorkommen und Stigmatisierungs‑, Scham- und Schuldgefühle bei den Frauen verstärken könnten. Es sollten *gut verständliche Informationen* über den *Ablauf eines Schwangerschaftsabbruchs* und die *gesetzlichen Regelungen, Adressen von Beratungsstellen und Praxen, evidenzbasierte Informationen über die (medizinischen) Folgen eines Schwangerschaftsabbruchs* (z. B. Umgang mit Ängsten, nicht mehr schwanger werden zu können) sowie *Informationen über Verhütung und sexuelle Aufklärung* zur Verfügung stehen.

##### Gleichberechtigte Zusammenarbeit und Beteiligung an Entscheidungen.

Evidenzbasierte und neutrale Informationen über die Möglichkeiten bei unbeabsichtigter Schwangerschaft wurden als entscheidende Grundlage für die Beteiligung an der Entscheidungsfindung genannt. Dazu gehört auch die Möglichkeit der *Wahl zwischen den Methoden*, was laut den Teilnehmenden in der Versorgungssituation in Deutschland nicht ausreichend möglich ist.

##### Einbezug von Familie und Freund:innen.

Es wurde über die Dimension der Einbeziehung von Partner:in, Familie und/oder Freund:innen von Frauen mit unbeabsichtigter Schwangerschaft in den Entscheidungsprozess und die Versorgung diskutiert. Die Möglichkeit einer Begleitung und eines Einbezugs in Beratungsangebote und die medizinische Versorgung wurde als wichtig für die Frauen befunden, jedoch unter Klärung der Präferenz der Frau und auch der Möglichkeit eines expliziten Nichteinbezugs.

##### Aktivierung von Personen in der Beratung/Behandlung.

Die Teilnehmenden kamen zu dem Schluss, dass das Empowerment der Frauen in ihrer Fähigkeit, für ihre Situation und ihr Leben eine *selbstbestimmte Entscheidung* zu treffen, ein Hauptziel in der Versorgung sein sollte.

##### Unterstützung des psychischen Wohlbefindens.

Entsprechend den Teilnehmenden sollten Versorgende das psychische Wohlbefinden der Frauen im Blick haben und unterstützen, zum Beispiel durch ausreichend Zeit, das Anbieten von Raum für Gefühle, Einfühlungsvermögen und vor allem das Ansprechen möglicher ambivalenter Gefühle (z. B. nach einem Abbruch gleichzeitig erleichtert und traurig zu sein). Die Teilnehmenden wiesen darauf hin, dass viele Frauen *Scham und Schuldgefühle* in der Situation eines Abbruchs empfinden.

##### Unterstützung des körperlichen Wohlbefindens.

Die Dimension der Unterstützung des körperlichen Wohlbefindens stand nicht im Fokus der Diskussionen. Dennoch diskutierten die Teilnehmenden über die Bedeutung des Angebots von Schmerzmitteln, da das Empfinden bei jeder Frau individuell sei.

##### Patient:innensicherheit.

Zuletzt wurde die Dimension Patient:innensicherheit diskutiert und betont, dass diese erhöht werden kann, zum einen durch das Angebot von medikamentösen Abbrüchen, da dadurch ein operativer Eingriff verhindert wird, zum anderen auch durch das Bereitstellen einer Notfalltelefonnummer im Fall von Komplikationen sowie durch den Schutz der Privatsphäre der Patientinnen z. B. im Praxisablauf.

#### Makroebene

##### Stigmatisierung der Betroffenen.

Die in die Workshops am intensivsten diskutierte Kategorie in dieser Ebene war die Stigmatisierung von Frauen mit einer unbeabsichtigten Schwangerschaft und einem Wunsch nach einem Schwangerschaftsabbruch. Hier wurde Stigmatisierung im ganzen Prozess der Versorgung, insbesondere auch bei der Feststellung der Schwangerschaft durch Gynäkolog:innen, die selbst keine Abbrüche durchführen, beschrieben. Dadurch würden Gefühle wie Scham und Schuld verstärkt und das Thema oft geheim gehalten. Um die *Entstigmatisierung und Enttabuisierung* von Schwangerschaftsabbrüchen zu fördern, sollten Abbrüche als normaler und wichtiger Teil der gynäkologischen Praxis wahrgenommen und eine objektive Behandlung von Frauen in dieser Notsituation gewährleistet werden. Die Versorgenden wiesen darauf hin, dass eine unbeabsichtigte Schwangerschaft jede Frau treffen kann, unabhängig von ihrer Bildung, ihrem sozioökonomischen Status oder ihrem (gebärfähigen) Alter.

##### Stigmatisierung der Beratenden/Behandelnden.

Des Weiteren wurde die Stigmatisierung von Versorgenden, welche Schwangerschaftsabbrüche anbieten, diskutiert. Die Teilnehmenden berichteten, dass einige Behandelnde, die Schwangerschaftsabbrüche anbieten, Ablehnung durch ärztliche Kolleg:innen erfahren.

##### Politische und rechtliche Aspekte.

Die Folgen der aktuellen politischen und rechtlichen Regelung auf eine personenzentrierte Versorgung von unbeabsichtigt Schwangeren wurden im Zusammenhang mit verschiedenen Dimensionen diskutiert. Dabei wurden die Auswirkungen des bis Juni 2022 bestehenden § 219a StGB auf die Informationslage beschrieben und die Notwendigkeit, evidenzbasierte Informationen sowie Adressen von Praxen und deren Angebot zugänglicher zu machen. Es wurde diskutiert, dass die verpflichtende Schwangerschaftskonfliktberatung nach § 219 als Zwang empfunden werden kann, wenn bereits eine Entscheidung für einen Schwangerschaftsabbruch getroffen wurde. Einige Versorgende beschrieben aufgrund des § 218 StGB (welcher Schwangerschaftsabbrüche als Straftatbestand definiert, der unter bestimmten Voraussetzungen nicht geahndet wird) das Gefühl zu haben, in einer „Grauzone“ zu arbeiten.

##### Corona-Pandemie.

In der Makroebene wurden auch die Einflüsse der Covid-19-Pandemie zusammengefasst. Hier wurden die in dieser Zeit nochmals verstärkten Hürden beim Zugang zu Praxen und Beratungsstellen beschrieben sowie, als positive Folge, das verstärkte Angebot und die Nutzung von telemedizinischer Schwangerschaftskonfliktberatung oder telemedizinisch begleiteten Schwangerschaftsabbrüchen.

## Diskussion und Ausblick zur CarePreg-Studie

Die Ergebnisse zur Perspektive von Versorgenden auf die Relevanz und aktuelle Umsetzung von Personenzentrierung in der psychosozialen und medizinischen Versorgung von Frauen mit Wunsch nach einem Schwangerschaftsabbruch in Deutschland zeigen, dass alle Dimensionen als ausgesprochen wichtig betrachtet wurden, jedoch zum jetzigen Stand noch nicht entsprechend den Empfehlungen der WHO und der S2k-Leitlinie zu Schwangerschaftsabbrüchen im ersten Trimenon umgesetzt sind. Zudem wurden weitere Faktoren wie die Stigmatisierung der Frauen und die politisch-rechtliche Lage als Hürden für eine hochwertige personenzentrierte Versorgung betrachtet.

Diese Ergebnisse stehen im Einklang mit internationalen Studien zu Barrieren in der Versorgung bei Schwangerschaftsabbruch [8, 12, 26]. In den meisten hoch entwickelten westeuropäischen Ländern gibt es für Frauen, die einen Abbruch wünschen, immer noch einen Mangel an wissenschaftlich fundierten Informationen über die Methoden und Abläufe eines Abbruchs und es besteht die Gefahr von irreführenden Informationen durch Abtreibungsgegner von Antiabtreibungsinitiativen [8]. Auch die Bereitschaft von Mediziner:innen, Schwangerschaftsabbrüche durchzuführen und damit den Zugang zu diesen zu verbessern, wird in internationalen Studien als limitiert durch rechtliche, politische, institutionelle und finanzielle Faktoren beschrieben, eine Stigmatisierung von Versorgenden von unbeabsichtigt Schwangeren wird ebenfalls beklagt [26, 27]. Die Möglichkeit des medikamentösen Schwangerschaftsabbruchs wird in internationalen Studien als eine weniger invasive, sichere und leicht zugängliche Methode beschrieben [28], jedoch wird das Wissen der Versorgenden zu dieser Methode als eher gering eingestuft [29]. Auch in Deutschland werden medikamentöse Schwangerschaftsabbrüche im Vergleich zu anderen Ländern in geringerem Maße angeboten und mit großen regionalen Unterschieden durchgeführt [30, 31]. Allerdings beschreibt die S2k-Leitlinie medikamentöse Abbrüche als gleichwertige Methode zu chirurgischen Abbrüchen und befürwortet den telemedizinischen Ansatz.

### Weitere, bisher nicht publizierte Ergebnisse der CarePreg-Studie

Nach der Untersuchung der Versorgungssituation aus Sicht der Versorgenden wurde in der 1. Phase der CarePreg-Studie auch die Perspektive der Betroffenen eingeholt. Hierzu wurden telefonische Interviews mit 34 Frauen mit einem Schwangerschaftsabbruch innerhalb der letzten 5 Jahre geführt und diese hinsichtlich ihrer positiven und negativen Erfahrungen in der Beratung und der medizinischen Versorgung befragt. Die Daten wurden ebenfalls mit dem Fokus auf die Dimensionen von Personenzentrierung inhaltsanalytisch ausgewertet. Insgesamt ließen sich große Übereinstimmungen mit der Perspektive der Versorgenden auf die Versorgungssituation feststellen. So wurde zum Beispiel auch von den Betroffenen der Zugang zur Versorgung besonders oft erwähnt, mit der besonderen Relevanz von zeitnahen Terminen, wohnortnahen Angeboten, Zugang zu Adressen von Praxen, die Schwangerschaftsabbrüche anbieten, Zugang zu Wahlmöglichkeiten der Methode und dem Wunsch der Kostenübernahme durch die Krankenkasse. Entsprechend den hier beschriebenen Ergebnissen der Workshops mit Versorgenden wurde auch von den Betroffenen selbst erwartete oder real erfahrene Stigmatisierung sehr häufig thematisiert.

In der anschließenden deutschlandeweiten Online-Befragung wurden insgesamt 240 Teilnehmende, die sich aktuell in der Situation einer unbeabsichtigten Schwangerschaft mit Wunsch nach einem Schwangerschaftsabbruch befanden oder innerhalb der letzten 2 Monate eine Schwangerschaft nach der Beratungsregel abgebrochen hatten, über 3 Erhebungszeitpunkte im Zeitraum von 12 Monaten befragt. Dabei wurden die erlebte Personenzentrierung in der psychosozialen und medizinischen Versorgung sowie unter anderem die psychische Belastung, die Zufriedenheit mit der Versorgung und mit der getroffenen Entscheidung über die Schwangerschaft als auch Stigmatisierungserwartungen und -erfahrungen erfragt. Hier zeigte sich insbesondere im Kontext der Beratung eine hohe bis sehr hohe Personenzentrierung für die Dimensionen des Integrativen Modells der Personenzentrierung. Der Bedarf an psychischer Unterstützung wurde aus Sicht etwa der Hälfte der Teilnehmenden nicht ausreichend adressiert, insbesondere bei hoher Belastung zum Zeitpunkt der Feststellung der Schwangerschaft und zum Zeitpunkt des Abbruchs. Die Sorge vor Stigmatisierung während der Beratung und der medizinischen Versorgung war hoch.

Diese weiteren Ergebnisse der CarePreg-Studie werden derzeit für die Veröffentlichung vorbereitet.

## Fazit

Die ersten Ergebnisse der CarePreg Studie zeigen, dass Personenzentrierung im Kontext der psychosozialen und medizinischen Versorgung bei unbeabsichtigt Schwangeren von hoher Relevanz ist. Die aktuellen regional bestehenden Hürden im Zugang zur Versorgung und zu Informationen zum Schwangerschaftsabbruch und entsprechenden Versorgungsstellen sowie Stigmatisierung sowohl innerhalb der Gesellschaft als auch speziell im Kontext der Versorgung limitieren die Personenzentrierung. Einen zusätzlichen Einfluss auf die geringe Qualität der Versorgung hat laut den befragten Versorgenden die aktuelle rechtliche Lage. Die Versorgungssituation sollte demnach weiter verbessert werden, um einen Standard entsprechend den Empfehlungen der WHO und der deutschen S2k-Leitlinie zu garantieren.
